# Perioperative factors determine outcome after surgery for severe acute pancreatitis

**DOI:** 10.1186/cc2991

**Published:** 2004-11-02

**Authors:** Jan J De Waele, Eric Hoste, Stijn I Blot, Uwe Hesse, Piet Pattyn, Bernard de Hemptinne, Johan Decruyenaere, Dirk Vogelaers, Francis Colardyn

**Affiliations:** 1 Intensivist, Intensive Care Unit, Ghent University Hospital, Gent, Belgium; 2 Researcher, Intensive Care Unit, Ghent University Hospital, Gent, Belgium; 3 Surgeon, Intensive Care Unit, Ghent University Hospital, Gent, Belgium; 4 Infectious Diseases Consultant, Department of Surgery, Ghent University Hospital, Gent, Belgium

**Keywords:** acute necrotizing pancreatitis, infected pancreatic necrosis, multiple organ failure, severe acute pancreatitis

## Abstract

**Introduction:**

There is evidence that postponing surgery in critically ill patients with severe acute pancreatitis (SAP) leads to improved survival, but previous reports included patients with both sterile and infected pancreatic necrosis who were operated on for various indications and with different degrees of organ dysfunction at the moment of surgery, which might be an important bias. The objective of this study is to analyze the impact of timing of surgery and perioperative factors (severity of organ dysfunction and microbiological status of the necrosis) on mortality in intensive care unit (ICU) patients undergoing surgery for SAP.

**Methods:**

We retrospectively (January 1994 to March 2003) analyzed patients admitted to the ICU with SAP. Of 124 patients, 56 were treated surgically; these are the subject of this analysis. We recorded demographic characteristics and predictors of mortality at admission, timing of and indications for surgery, and outcome. We also studied the microbiological status of the necrosis and organ dysfunction at the moment of surgery.

**Results:**

Patients' characteristics were comparable in patients undergoing early and late surgery, and there was a trend toward a higher mortality in patients who underwent early surgery (55% versus 29%, *P *= 0.06). In univariate analysis, patients who died were older, had higher organ dysfunction scores at the day of surgery, and had sterile necrosis more often; there was a trend toward earlier surgery in these patients. Logistic regression analysis showed that only age, organ dysfunction at the moment of surgery, and the presence of sterile necrosis were independent predictors of mortality.

**Conclusions:**

In this cohort of critically ill patients operated on for SAP, there was a trend toward higher mortality in patients operated on early in the course of the disease, but in multivariate analysis, only greater age, severity of organ dysfunction at the moment of surgery, and the presence of sterile necrosis, but not the timing of the surgical intervention, were independently associated with an increased risk for mortality.

## Introduction

Morbidity and mortality after surgery for severe acute pancreatitis (SAP) remain considerable, despite the introduction of new strategies to reduce infectious complications [1,2], such as antibiotic prophylaxis, early enteral nutrition [3], and the recognition of complications such as abdominal compartment syndrome in severely ill patients [4].

There is limited evidence in the literature that postponing surgery beyond the initial phase of the disease leads to improved survival. Mier and colleagues [5] randomized 36 patients to early versus late surgery, and stopped the study after an interim analysis showed that patients operated on early had a higher mortality. This finding has been confirmed by others in retrospective studies. Hungness and colleagues [6] found a trend toward an increased mortality in 14 of 26 patients who were operated on within the first two weeks of diagnosis. Hartwig and colleagues [7] found in a review of 62 surgically treated patients that those operated on within three days had a higher mortality rate (53% versus 22%, *P *= 0.02). In contrast, Fernández-del Castillo and colleagues [8] found a similar mortality rate in their patients when either operated on early or later than 6 weeks after admission. There are conflicting data on the impact of timing of surgery on mortality, and the different definitions used for early surgery, ranging from three days to six weeks, makes comparing the data in the literature difficult.

All studies that reported increased mortality in patients undergoing early surgery included patients operated on for a range of indications (such as absence of clinical improvement after 3–5 days, persistent pancreatitis, infected necrosis, pancreatic abscess and sepsis syndrome) at different stages of the disease. It is not clear to what extent the severity of illness at the moment of surgery or the microbiological status of the necrosis were confounding factors and were a bias in finding increased mortality rates for early surgery.

In this paper we report our study on the impact of the timing of surgical intervention and perioperative factors (severity of organ dysfunction and microbiological status of the necrosis) on mortality in patients undergoing surgery for SAP.

## Materials and methods

### Data collection

We retrospectively (January 1994 to March 2003) analyzed all patients admitted with SAP to the intensive care unit (ICU) of the Ghent University Hospital, a tertiary referral centre with a total of 1060 beds. SAP was defined in accordance with the criteria described by the International Symposium on Acute Pancreatitis [9]. Patients were identified from the hospital registry with the use of the International Classification of Diseases (ICD-9-CM) code for acute pancreatitis' (577.0). Preoperative data collected included age, sex, etiology, use of antibiotics, C-reactive protein level, Ranson score and Acute Physiology And Chronic Health Evaluation (APACHE) II score [10] on admission. Time to the first surgical intervention, the severity of organ dysfunction at the day of the first surgical intervention (as assessed by the sepsis-related organ failure assessment (SOFA) score [11]), length of stay in the ICU and in the hospital, and mortality were retrieved from the patient's file.

The occurrence of organ dysfunction during the ICU stay was recorded, and organ dysfunction was defined as follows (based on a score of 2 or more in the SOFA scoring system): (1) cardiovascular dysfunction was defined as hypotension requiring vasoactive medication; (2) renal dsyfunction, serum creatinine above 2.0 mg/dl; (3) respiratory dyscfunction, the need for mechanical ventilation or a PaO_2_/FIO_2 _ratio of less than 300.

Microbiological data collected included peroperative cultures from the initial surgical intervention, and fine-needle aspirates (FNAs), when available. Infected pancreatic necrosis was defined as the presence of microorganisms in cultures obtained at the first operation or in cultures of a FNA of the pancreatic necrosis without previous surgery; consequently, sterile pancreatic necrosis was defined as negative cultures from intraoperative cultures, independently of infections occurring later in the course of the disease.

Mortality was defined as in-hospital mortality.

The study was approved by the local ethical committee.

### Study design

Patients treated surgically early in the course of the disease were compared with patients who underwent delayed surgical intervention. Early surgery was defined as surgery within 12 days of diagnosis, as described in the prospective trial by Mier and colleagues [5]. Furthermore, we compared patients with sterile pancreatic necrosis with patients with infected necrosis, and survivors with non-survivors, using univariate and multivariate analysis techniques.

### Patient management

All patients were admitted to the ICU before or after surgical treatment and were treated by the same surgical team. The use of antibiotic prophylaxis was left to the discretion of the attending ICU physician. Enteral nutrition was started as early as possible. Computed tomography (CT) scanning and FNA of the pancreatic necrosis was performed on an individual patient base, namely when the clinical condition of the patient was suggestive of infection of the pancreatic necrosis. Indications for surgery were a documented infection of pancreatic necrosis (as evidenced by positive cultures from FNA), a deterioration of the clinical condition of the patient, unresolving pancreatitis or suspected pancreatic infection without proof on FNA or CT scan. Surgical intervention consisted of necrosectomy through a midline laparotomy as described by Beger and colleagues [12]. The pancreas was debrided using blunt dissection, and two to four large-calibre drains were inserted in the retroperitoneum. Continuous postoperative lavage of the retroperitoneum was started initially at a rate of 500–1000 ml/h, and progressively decreased, on the basis of the general condition of the patient, inflammatory parameters (C-reactive protein), and the macroscopic aspect of the drain effluent.

### Statistical analysis

Statistical analysis was performed with SPSS for Windows 11.0.1^® ^(SPSS, Chicago, IL, USA). Continuous variables were compared by using Student's *t*-test or the Mann–Whitney *U*-test where appropriate. Categorical data were compared with the ?^2 ^or Fisher Exact test. A double-sided *P *value of less than 0.05 was considered statistically significant. Parameters found to be different in survivors and non-survivors in univariate analysis with a *P *value of 0.25 or less were entered in a logistic regression model with mortality as the dependent variable, to identify factors available at the moment of surgery that were independently associated with mortality.

## Results

### Patients

Of 124 patients with SAP, 56 (35 male, 21 female) were treated surgically. The mean age of the patients was 56 years (SD 13.5). The cause of the pancreatitis was biliary tract stones in 19 patients (33.9%), alcohol in 21 (37.5%), trauma in 6 (10.7%), hyperlipemia in 1 (1.8%) and idiopathic in 9 (16.1%). Thirty-nine patients (69.6%) were referred from other hospitals; for three patients the first surgical intervention was performed in the referring hospital 1 day before referral (*n *= 2) or on the day of referral (*n *= 1).

### Early versus late surgical intervention

Twenty-two patients (39.2%) were operated on within the first 12 days of diagnosis of pancreatitis (median 5 days, interquartile range 3–9), and 34 (60.8%) later than 12 days after admission (median 20 days, interquartile range 17–31).

Age and gender distribution were comparable in both groups (Table 1). Disease severity, assessed by Ranson and APACHE II scores on admission in these patients, was not different; neither was the SOFA score at the day of surgery. Indications for surgery in patients operated on early were different from those operated on later in the course of the disease. In patients operated on early, deterioration of multiple organ dysfunction syndrome (MODS) was the indication for surgical intervention in 41% of the patients, compared with 9% in the late surgery group. Overall, the length of stay in the hospital was significantly longer for the patients who underwent surgery late in the course of the disease, even after censoring the patients who died in both groups. Duration of ICU stay was not different. There was a trend toward a higher mortality in the early surgery group (55% versus 29%, *P *= 0.06).

### Microbiological status of necrosis and mortality

In 26 (46.4%) patients, intraoperative cultures confirmed the diagnosis of infected pancreatic necrosis. Microorganisms isolated from the necrosis are listed in Table 2. Gram-negative and Gram-positive microorganisms were present in comparable numbers (38.9%); seven patients had fungal infections at the first operation. In 10 patients more than one organism was isolated. Thirty of 56 patients (54%) had sterile pancreatic necrosis at the moment of the first surgical intervention. Patient characteristics, severity of disease, and the timing of surgery were not different in patients with sterile or infected pancreatic necrosis (Table 3). There was a trend toward a higher occurrence rate of organ failure in patients with sterile pancreatic necrosis, and mortality was significantly higher in patients with sterile necrosis (57%) than in patients with infected necrosis (19%) (*P *= 0.004).

Especially in patients undergoing early surgery, mortality was significantly higher in patients with sterile pancreatic necrosis (85% versus 11%, *P *= 0.001) In the patients who underwent delayed surgery, there was no difference in mortality between patients with sterile pancreatic necrosis and those with infected pancreatic necrosis (35% in patients with sterile pancreatic necrosis and 23% in patients with infected pancreatic necrosis, *P *= 0.71).

### Factors influencing outcome after surgical intervention

Overall mortality in our patients was 39.2% (22 of 56 patients). Table 4 summarizes differences between survivors and non-survivors. In univariate analysis, patients who died were older, had higher APACHE II scores on admission, higher SOFA scores on the day of surgery, more often sterile necrosis, and more often organ dysfunction during their ICU stay, and were operated more often because of MODS. There was also a trend toward earlier surgical intervention in patients who died.

The following variables were entered in a logistic regression analysis: age, SOFA score on the day of surgery, the presence of sterile pancreatic necrosis at surgery, and interval from diagnosis to surgical intervention as a continuous variable. SOFA score at the day of surgery was preferred to APACHE II score on admission and deteriorating MODS as an indication for surgery, because it better describes the severity of illness at the moment of surgery, and the difference in univariate analysis was more significant. In multivariate analysis, only age, SOFA score at the moment of surgery, and the presence of sterile necrosis were associated with mortality (Table 5).

## Discussion

It has been suggested that postponing surgery beyond the initial phase of the disease leads to improved survival [5-7]. In this analysis of 56 patients undergoing surgery because of SAP, we found that disease severity at the moment of surgery, age, and the presence of sterile necrosis, but not early surgery, determined mortality. The trend toward an increased mortality in patients operated on within 12 days of diagnosis, found in univariate analysis, was apparently confounded by perioperative factors.

Disease severity at admission has long been recognized as an important factor determining outcome in patients with SAP, irrespective of surgical intervention. So far, Ranson score at admission and C-reactive protein levels at 48 hours [13,14] have proven to be the best predictors of disease severity; more recently, the APACHE II score [15] and determination of the individual Ranson parameters [16] at 48 hours showed improved predictive value compared with admission scores.

In patients undergoing surgery for SAP, perioperative organ dysfunction affects outcome. Connor and colleagues [17] reported that a high postoperative APACHE II score was the only factor associated with mortality in a group of moderately ill patients (initial APACHE II score 9) undergoing pancreatic necrosectomy. Hungness and colleagues [6] reported higher organ failure scores and more advanced age in patients who died after surgery for SAP. The present study further confirms these findings.

The reason for this increased mortality is not clear. Surgical intervention by itself in the early phase of the disease is a possible explanation for the high mortality rate in patients undergoing early surgery, and has been suggested by several authors [6,7], but the evidence for this is indirect. It seems plausible that in the early stage of the disease, when there is peripancreatic and retroperitoneal inflammation, surgery is often difficult, with increased blood loss. Patients with severe organ dysfunction might also be more prone to other complications that could arise from the surgical intervention, such as gastrointestinal ischemia or blood loss. Another possibility is that in these patients other complications – that have only recently been recognized – were involved, and were left untreated for too long. Intra-abdominal hypertension and abdominal compartment syndrome are increasingly described in patients with SAP [4,18], and can lead to multiple organ dysfunction. Other problems such as relative adrenal insufficiency [19], which is increasingly recognized in patients with septic shock [20] or high-risk surgical patients [21], might be involved.

The fact that sterile necrosis is a risk factor for mortality in patients undergoing surgery is an important finding. At first sight this might be in sharp contradiction of the fact that infected pancreatic necrosis has been associated with increased mortality in several studies. It should be kept in mind that this undoubtedly is true for patients with SAP as a whole, and that this analysis included only patients who were operated on.

The findings of the present study are in line with the current concept that patients with sterile pancreatic necrosis do not need surgery, although this is still advocated by some experts in the field. Several authors have reported mortality rates below or about 10% when managing these patients non-operatively [22-24]. Le Mée and colleagues reported that, in most of their patients, organ dysfunction was reversible if necrosis remained sterile [25]. These and our results suggest that in patients with suspected infection of the pancreatic necrosis, the presence of microorganisms should be actively sought with ultrasound-guided or CT scan-guided FNA before surgical intervention is considered [26].

Our study could not reproduce the negative impact of early surgery on outcome after adjustment for other factors that were associated with increased mortality in univariate analysis. The often-used strategy to postpone surgery in patients with SAP is based on limited data. Mier and colleagues [5] randomized 36 patients with SAP to early (within 48–72 hours) versus late surgery (later than 12 days). Mortality in the group that underwent early debridement was 56%, 3.4-fold that in the control group, a result that halted the trial.

This finding has also been reported by other investigators, but the definition of early surgery should be carefully considered, because the use of different time frames makes it very difficult to compare the evidence available in the literature. Fernandez-del Castillo and colleagues [8] analyzed 64 patients operated on with a technique of closed packing, and found that mortality in patients operated on within the first six weeks after onset of the disease was not different from mortality in patients operated on later than six weeks. This study included patients with pancreatic abscesses, a disease that has a different clinical course and prognosis from that of patients who require surgery for infected pancreatic necrosis. Patient selection and the definition of early surgery make it very difficult to compare this study with ours.

Hartwig and colleagues [7] found a significantly higher mortality in patients operated on within 72 hours (53% versus 22%, *P *= 0.02) in 136 patients treated between 1980 and 1997, about half of them surgically. During the study period, indications for surgery gradually shifted from a lack of clinical improvement after 2–3 days to a suspicion of infected necrosis, resulting in patients being operated on later, and lower mortality rates. Over all, operating less, and if necessary, as late as possible, markedly improved outcome.

From the data available in the literature, the advice to postpone surgery by default beyond the first 2–3 weeks seems to be based on unblinded, unadjusted, or retrospective analyses. A similar process has been observed with the use of prophylactic antibiotics. The use of these became widespread on the basis of limited evidence, but the benefit could not be demonstrated in a controlled randomized trial [27]. Although we agree that there are several pathophysiological considerations in deferring surgery, such as those described above, we did not find any evidence that the timing of surgery by itself influenced outcome.

## Conclusion

Our data suggest that not the timing of the surgical intervention, but rather perioperative factors, determine mortality in critically ill patients undergoing necrosectomy for SAP. We found that mortality was associated with greater age, increasing severity of organ dysfunction, as expressed by the SOFA score at the moment of surgery, and the presence of sterile necrosis. In future studies on the effect of timing of surgery, the severity of organ dysfunction and microbiological status at surgery should be evaluated as possible confounding variables.

## Key messages

• In a series of 56 patients who were treated surigically for severe acute pancreatitis, no effect of the timing of surgery was found if perioperative factors such as severity of illness and microbiological status of the necrosis were considered.

## Abbreviations

APACHE = Acute Physiology And Chronic Health Evaluation; CT = computed tomography; FNA = fine-needle aspirate; ICU = intensive care unit; MODS = multiple organ dysfunction syndrome; SAP = severe acute pancreatitis; SOFA = sepsis-related organ failure assessment.

## Competing interests

The author(s) declare that they have no competing interests.

## Authors' contributions

JDW, UH and FC were responsible for the conception and design of the study. JDW and SB acquired a substantial portion of the data. JDW, EH and DV performed the analysis and interpretation of data. JDW and DV drafted the manuscript. FC, PP, BDH, JDC and EH undertook critical revision of the manuscript for important intellectual content. EH and SB were responsible for statistical expertise. FC performed supervision and took overall responsibility for all aspects of the project or study. All authors read and approved the final manuscript.

## Figures and Tables

**Table 1:** Characteristics, indications for surgery and outcome of patients operated on for severe acute pancreatitis (*n *= 56)

Parameter	Early surgery^a ^(*n *= 22)	Late surgery^a ^(*n *= 34)	*P*
Patient characteristics			
Age, years (mean ± SD)	54 ± 14.8	56 ± 12.8	0.58
Male sex	16 (72.7%)	19 (55.9%)	0.20
APACHE II score (mean ± SD)	22 ± 12.1	19 ± 8.9	0.47
Ranson score (mean ± SD)	6.2 ± 2.46	5.8 ± 1.80	0.59
Sterile necrosis at first surgical intervention	13 (59.1%)	17 (50%)	0.50
Interval from diagnosis to surgery, days (median and IQR)	5 (3–9)	20 (17–31)	<0.001
SOFA score at surgical intervention (median and IQR)	4 (2–8)	4 (2–8)	0.78
Indications for surgery			
Documented infection of pancreatic necrosis	5 (22.7%)	14 (41.2%)	0.25
Deteriorating clinical condition	9 (40.9%)	3 (8.8%)	0.007
Unresolving pancreatitis or suspected infection of pancreatic necrosis	8 (36.4%)	17 (50%)	0.41
Outcome			
LOS in ICU, days (median and IQR)	14 (5–33)	14 (6–35)	0.75
LOS in hospital, days (median and IQR)	29 (15–58)	87 (54–106)	<0.001
LOS in ICU in hospital survivors, days (median and IQR)^b^	16 (4–46)	12 (5–31)	0.92
LOS in hospital in hospital survivors, days (median and IQR)^b^	44 (30–107)	88 (60–106)	0.034
Mortality	12 (54.5%)	10 (29.4%)	0.06

APACHE II score, Acute Physiology And Chronic Health Evaluation II score; ICU, intensive care unit; IQR, interquartile range; LOS, length of stay; SOFA, sepsis-related organ failure assessment.

^a^Early surgery was defined as surgery within the first 12 days after admission.

^b^Early surgery, *n *= 10; late surgery, *n *= 24.

**Table 2:** Microorganisms (*n *= 36) isolated from 26 patients with infected pancreatic necrosis

Microorganism	*n*
Gram-positive bacteria	14
*Staphyloccus epidermidis*	4
*Staphylococcus aureus*	4
Enterococci	6
Gram-negative bacteria	14
*Escherichia coli*	7
*Enterobacter aerogenes*	4
*Pseudomonas *spp.	1
*Stenotrophomonas *sp.	1
*Proteus mirabilis*	1
Fungi	7
*Candida *spp.	7
Anaerobes	1
Bacteroides	1

**Table 3:** Comparison of patients with infected and sterile pancreatic necrosis

Characteristic	Infected pancreatic necrosis (*n *= 26)	Sterile pancreatic necrosis (*n *= 30)	*P*
Age, years (mean ± SD)	56 ± 13.6	55 ± 13.7	0.75
Male gender	18 (78%)	17 (57%)	0.33
APACHE II score (mean ± SD)	20 ± 9.7	21 ± 11.1	0.71
CRP at admission, mg/dl (mean ± SD)	17 ± 14.7	16 ± 15.8	0.87
Ranson score (mean ± SD)	6.2 ± 1.7	5.7 ± 2.3	0.50
Organ failure			
Respiratory insufficiency	19 (7%)	24 (80%)	0.54
Acute renal failure	15 (58%)	24 (80%)	0.07
Cardiovascular failure	21 (81%)	20 (67%)	0.12
Interval from diagnosis to surgery, days (median and IQR)	14 (6–24)	18 (6–31)	0.36
LOS in ICU, days (median and IQR)	14 (5–32)	12 (5–30)	0.86
LOS in hospital, days (median and IQR)	68 (44–90)	54 (19–97)	0.36
Mortality	5 (19%)	17 (57%)	0.004

APACHE II score, Acute Physiology And Chronic Health Evaluation II score; CRP, C-reactive protein; ICU, intensive care unit; IQR, interquartile range; LOS, length of stay.

**Table 4:** Comparison of survivors and non-survivors (*n *= 56)

Characteristic	Non-survivors (*n *= 22)	Survivors (*n *= 34)	*P*
Age, years (mean ± SD)	62 ± 12.0	51 ± 12.8	0.002
APACHE II score (mean ± SD)	25 ± 8.5	18 ± 10.6	0.019
Ranson score (mean ± SD)	6.3 ± 2.03	5.7 ± 2.09	0.36
Male gender	15 (68%)	20 (59%)	0.48
CRP at admission, mg/dl (mean ± SD)	176 ± 162.3	170 ± 142.2	0.90
Organ dysfunction			
Respiratory insufficiency	22 (100%)	21 (62%)	0.001
Acute renal failure	22 (100%)	17 (50%)	<0.001
Cardiovascular failure	20 (91%)	12 (35%)	<0.001
Sterile necrosis	17 (77%)	13 (38.2%)	0.004
SOFA score at surgery (median and IQR)	4 (9–13)	2 (2–7)	0.005
MODS as indication for surgery	8 (36%)	4 (12%)	0.045
Early surgical intervention	12 (55%)	10 (29%)	0.06
Interval from diagnosis to surgery, days (median and IQR)	11 (4–22)	18 (12–29)	0.09

APACHE II score, Acute Physiology And Chronic Health Evaluation II score; CRP, C-reactive protein; IQR, interquartile range; MODS, multiple organ dysfunction syndrome; SOFA, sepsis-related organ failure assessment.

**Table 5:** Multivariate analysis

Variable	*P*	OR	95% CI
Sterile necrosis	0.012	13.704	1.778–105.602
SOFA score at surgery (per point)	0.009	1.351	1.076–1.695
Age (per year older)	0.004	1.124	1.037–1.218
Interval from diagnosis to surgery	0.868	1.006	0.939–1.078

CI, confidence interval; OR, odds ratio; SOFA, sepsis-related organ failure assessment.
